# A System for Real-Time Detection of Abandoned Luggage

**DOI:** 10.3390/s25092872

**Published:** 2025-05-02

**Authors:** Ivan Vrsalovic, Jonatan Lerga, Marina Ivasic-Kos

**Affiliations:** 1 Faculty of Informatics and Digital Technologies, University of Rijeka, 51000 Rijeka, Croatia; ivan.vrsalovic@inf.uniri.hr; 2 Faculty of Engineering, University of Rijeka, 51000 Rijeka, Croatia; jonatan.lerga@riteh.uniri.hr; 3 Centre for Artificial Intelligence, University of Rijeka, 51000 Rijeka, Croatia

**Keywords:** computer vision, deep learning, object detection, YOLOv8, YOLOv11, DETR encoder–decoder transformer, OpenCV, luggage detection, surveillance

## Abstract

**Highlights:**

**What are the main findings?**

- The fine-tuned model based on YOLOv11-l architecture achieves better results in detecting people and luggage in surveillance camera footage than the YOLOv8 and DETR models fine-tuned on the same dataset.
- The fine-tuned YOLOv8 and YOLOv11 models in m and l versions significantly improve object detection accuracy in demanding surveillance scenes with many small and medium-sized objects, with mAP@0.5 from 3.34% to over 86%.
- The fine-tuned YOLOv11-l model shows excellent performance in object detection, with mAP@0.5 accuracy of 96% for medium-sized objects and 85% for small objects.
- An algorithm for detecting abandoned luggage in public areas in real-world scenes was designed, implemented in Python 3.10, and tested on different scenarios in airport scenes.
- Image datasets were created, with images collected from surveillance cameras in public areas of airports and walkways and prepared for machine learning of object detectors.

**What is the implication of the main finding?**

- The accurate detection of people and luggage significantly contributes to increasing the functionality of the abandoned luggage detection algorithm and creating a system that helps in monitoring public spaces and increasing safety in crowded public areas.
- Including adjustable parameters in the algorithm (such as luggage dwell time, owner’s distance from luggage) reduces false alarms and improves the system efficiency.

**Abstract:**

In this paper, we propose a system for the real-time automatic detection of abandoned luggage in an airport recorded by surveillance cameras. To do this, we use an adapted YOLOv11-s model and a proposed algorithm for detecting unattended luggage. The system uses the OpenCV library for the video processing of the recorded footage, a detector, and an algorithm that analyzes the movement of a person and their luggage and evaluates their spatial and temporal relationships to determine whether the luggage is truly abandoned. We used several popular deep convolutional neural network architectures for object detection, e.g., Yolov8, Yolov11, and DETR encoder–decoder transformer with a ResNet-50 deep convolutional backbone, we fine-tuned them on our dataset, and compared their performance in detecting people and luggage in surveillance scenes recorded by an airport surveillance camera. The fine-tuned model significantly improved the detection of people and luggage captured by the airport surveillance camera in our custom dataset. The fine-tuned YOLOv8 and YOLOv11 models achieved excellent real-time results on a challenging dataset consisting only of small and medium-sized objects. They achieved real-time precision (mAP) of over 88%, while their precision for medium-sized objects was over 96%. However, the YOLOv11-s model achieved the highest precision in detecting small objects, corresponding to 85.8%, which is why we selected it as a component of the abandoned luggage detection system. The abandoned luggage detection algorithm was tested in various scenarios where luggage may be left behind and in situations that may be potentially suspicious and showed promising results.

## 1. Introduction

Video surveillance systems are widely deployed in public areas such as airports, train stations, metro stations, shopping centers, banks, and schools, as well as other high-traffic locations to ensure the safety of people, infrastructure, and equipment while maintaining the uninterrupted operation of these facilities.

Among the security risks in public spaces like airports, unattended luggage poses a significant threat to public safety and can disrupt normal operations. Detecting abandoned luggage or identifying suspicious items left behind is a complex task due to the crowded nature of these environments, where large numbers of people are constantly entering, moving in various directions, and exiting while handling or standing near different types of luggage.

Traditional surveillance methods rely on extensive networks of cameras monitored by human operators who must maintain constant focus to identify and respond to potential threats. However, human operators are prone to fatigue and decreased concentration over time, which increases the likelihood of errors or missed detections. As a result, large teams are often required to ensure security, which drives the demand for automated security monitoring systems that can enhance efficiency and accuracy.

In recent years, automated approaches for detecting abandoned luggage within a camera’s field of view have been actively explored. Modern object detection algorithms, such as those from the YOLO family, have significantly advanced the real-time detection capabilities in diverse real-world scenarios. Earlier methods relied on traditional computer vision techniques like feature extraction combined with classifiers (e.g., SVM), background suppression, and motion analysis. However, these approaches struggle in dynamic environments due to their sensitivity to factors such as lighting changes, crowd density, viewing angles, and complex backgrounds.

This study evaluates popular deep learning architectures for object detection, including YOLOv8, YOLOv11 (Ultralytics LLC, Frederick, MD, USA), and the DETR (Meta Platforms, Inc., Menlo Park, CA, USA) encoder–decoder transformer with a ResNet-50 backbone, fine-tuned for domain adaptation on a custom surveillance dataset to detect people and luggage in crowded environments. Performance comparisons revealed that all fine-tuned models showed significant improvements in detection performances for these scenarios. Both YOLOv8 and YOLOv11 achieved excellent real-time results on a challenging dataset comprising only small and medium-sized objects. However, YOLOv11-s, although one of the smaller versions of the model, showed the best results in detecting small objects, such as luggage in our case, and the best accuracy, which makes it the most suitable algorithm for our detection system.

The main contributions of this research are as follows:

- Creation of a custom dataset CCTV-KD, capturing diverse real-world public-space scenarios in Korzo pedestrian zone and Dusseldorf airport with annotation of people and luggage, ready for training object detectors.
- Fine-tuned YOLOv8, YOLOv11, and DETR models, domain-optimized and tailored for detecting people and luggage in surveillance footage.
- Model performance analysis of fine-tuned YOLOv8 and YOLOv11 variants on complex scenes dominated by small and medium-sized objects captured from bird’s-eye view.
- Domain-optimized YOLOv11-s model achieving real-time precision (mAP) of 88%, with 93.4% mAP for medium objects and 85.8% mAP for small objects in demanding surveillance environments.
- A novel abandoned luggage detection algorithm combining temporal and spatial analyses of object tracking to infer luggage ownership and reliably identify abandoned items.

The paper starts with an overview of the field, then proceeds in Section 3 to detail the proposed system and the algorithm for abandoned luggage detection, focusing on addressing key limitations of traditional methods and providing effective solutions for real-world security challenges. In Section 4, key phases of the development of abandoned luggage systems are presented, followed by a description of custom model fine-tuning in Section 5, of the custom datasets in Section 6, and of the experiment setup in Section 7, and an evaluation of the results in Section 8. The implementation of the proposed algorithm and different scenarios with abandoned luggage are discussed in Section 9. The paper ends with the Discussion and Conclusions.

## 2. Related Work

The task of detecting abandoned luggage in video surveillance can be divided into two main phases: the first involves identifying both the luggage and its owner in a video frame, while the second assesses the likelihood that the luggage is truly abandoned based on the gathered information. Traditional approaches to luggage and owner detection frequently rely on background subtraction methods, which are straightforward to implement, yet ill-suited for complex scenes with numerous moving objects [1]. One of the earlier approaches utilized a Markov chain Monte Carlo model with Bayesian networks for luggage detection [2]. Although effective under controlled conditions, this method was limited in adapting to more complex and dynamic scenarios. The analysis of background changes over time was applied in [3] to detect stationary objects, a technically interesting approach but extremely sensitive to variations in lighting and camera vibrations, leading to a high false-positive rate. In [4], foreground segmentation was used to identify stationary objects and generate candidates for abandoned luggage, thereby reducing the number of false positives. Nevertheless, it was not sufficiently robust in real-world dynamic environments.

More advanced approaches have integrated traditional methods with convolutional neural networks (CNNs), which are adapted to a broad range of computer vision tasks such as classification, object detection, and image segmentation [2]. In [5], a hybrid approach was introduced, combining background subtraction for detecting static objects with a CNN for identifying abandoned luggage. This method achieved significant performance in controlled environments, reducing detection errors and improving accuracy. However, the computational requirements of such solutions remain a challenge in real-world applications.

Modern approaches rely on deep CNNs that are well-suited for a wide range of computer vision tasks [2] and, in this context, are used for the detection or tracking of images or videos captured by surveillance cameras. Briefly, detectors can be categorized into one-stage and two-stage models. In one-stage models such as YOLO (You Only Look Once), the entire detection process (localization and classification) takes place in a single pass through the network [2]. In contrast, two-stage approaches (e.g., the Faster R-CNN) first generate regional proposals and then perform classification and coordinate regression, which leads to higher computational demands and longer processing times [6]. Due to the real-time inference capabilities and relatively high detection accuracy in complex and dynamic scenarios, YOLO-based models (e.g., YOLOv5 and YOLOv8) are increasingly employed in security systems [4,7,8,9,10]. A summary of relevant research is presented in Table 1.

Also, it should be noted that surveillance scenes typically feature numerous small objects that occupy only a few pixels in each image. This characteristic presents two main challenges, i.e., difficulties in distinguishing small objects from background noise and increased susceptibility to occlusion, so that the justified requirements for the model implemented in the surveillance system are real-time performance capabilities and proficiency in detecting small and medium-sized objects. These features are essential for effective surveillance, ensuring that the system can accurately identify and track the objects of interest, even when they are small or partially obscured, while maintaining the speed necessary for real-time monitoring and responses. But, as noted in [12], many CNN models still struggle with this very problem, as during down-sampling, the CNN-based architecture loses critical spatial details. However, it was showed that Faster R-CNN was the top-performing architecture for small object detection, and YOLO was the second-best option [12] but with a significant speed advantage, which makes the YOLO model far more suitable for real-time applications, such as in surveillance systems, where rapid detection is critical. For this reason, in our experiments, we also selected the best YOLO models that achieved excellent performance in real time, with an emphasis on the detection of small objects.

## 3. Proposed Luggage Detection System

The proposed abandoned baggage detection system uses video footage collected from CCTV cameras at an airport, a customized deep CNN model for the real-time automatic detection of people and baggage in surveillance camera images, and the defined algorithm for recognizing abandoned baggage and raising an alarm to a security team.

The assumption is that some people may have one or more pieces of luggage, while others may be without luggage. Also, luggage does not move around the airport building on its own, being either pulled or carried by a person or transported by a person on a vehicle or train. Therefore, it is possible that someone could bring some luggage to a location, and if the luggage is left unattended, it is considered abandoned.

Prior to developing the algorithm for recognizing abandoned luggage, it is necessary to define rules that determine when and under what conditions luggage is considered abandoned and a potential security threat.

A. Definition of Abandoned Luggage

For luggage to be classified as abandoned in the proposed system, two criteria must be met:

1. **The luggage must be unattended**—all individuals who were within a predefined radius around the luggage at the time of its initial detection must have moved out of that radius.
2. **The luggage must remain in the same location for a specified period.**

When both conditions are satisfied, i.e., the luggage is left unattended outside a predefined radius and has remained stationary for a certain time greater than the duration set in the system, then the system marks the luggage as abandoned. These conditions are set to include all possible cases that may exist involving people, the owner of the luggage, and the luggage, and to reduce the number of false positive detections.

For instance, if a person is waiting in a queue with luggage placed in a certain spot for some time while remaining nearby, the luggage is presumed to be supervised, linked to that individual, and therefore neither abandoned nor identified as suspicious.

**Figure 1:**
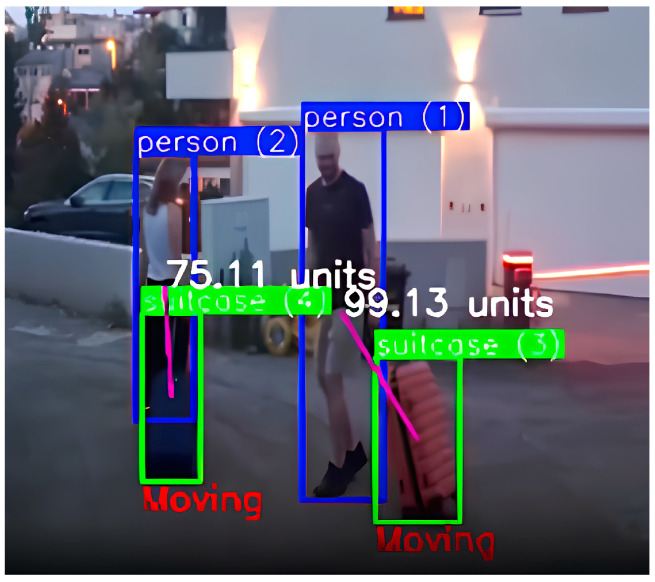
Group detection with the detection of 2 people marked with blue bounding boxes and two suitcases marked with green bounding boxes. In order for the luggage not to be marked as abandoned, it is important that it is close to its owner or that it is moving, so the image shows the calculated distance between the person and the suitcase and the label “Moving” as the movement of the person and suitcase is detected.

It is also possible for an entire group to move together, with some or all members carrying their own luggage (Figure 1). If one or several members of the group leave while leaving their luggage behind with the others, the luggage is still considered under the group’s supervision. Consequently, such a scenario would not be flagged as suspicious.

B. System and Algorithm for Abandoned Luggage detection

**Figure 2:**
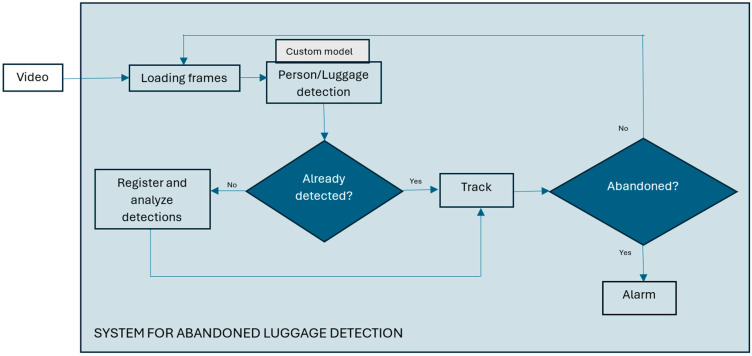
System for abandoned luggage detection.

The system components for abandoned luggage detection are shown in Figure 2. The system loads video stream from a surveillance camera and then takes frames from the video every 0.25 s in order not to lose useful information from the footage and to speed up image analysis. Each extracted image is the input to a customized object detector that detects all people and luggage in the input image that is in the camera’s field of view. For tracking the detected objects across frames, we used YOLOv11’s default multi-object tracking algorithm, ByteTrack. ByteTrack works by associating detections frame by frame, keeping high-confidence detections while also considering low-confidence ones to improve tracking stability. This approach ensures better object identity persistence and reduces the tracking errors in complex environments, enabling a robust monitoring of luggage movement and ownership status [13]. It is crucial for the detection model to accurately identify people and luggage in real time with a high response rate, ensuring that no potentially suspicious objects go undetected.

The system then uses an algorithm to identify abandoned luggage based on an analysis of the spatial and temporal dynamics of the luggage and on identifying situations in which the luggage has been left unattended. The first processing step involves the initial detection of people and luggage, assigning each detected object a unique ID. A limit was set according to which the size of the objects to be detected and tracked should be greater than 20 pixels, because very distant objects that occupy only a few pixels cannot be successfully detected in an image, and it is assumed that they will be better visible using another camera that is closer to them. This limit is specific to the system and can be adjusted according to the parameters of each system, considering the limitations of the detector.

Then, the system checks which people are within a defined radius of the luggage and maps that luggage to one or more people nearby. For each subsequent frame, the system checks whether the persons initially detected near the luggage are still within its radius, meaning whether the luggage remains under their supervision.

The pseudocode of Algorithm 1 is written below:
**Algorithm 1**: Abandoned luggage identification**INITIALIZATION:**   - Load tracking model 
   - Define parameters:
      - Threshold time (T_threshold) 
      - Ownership radius (R_own) 
      - Movement Threshold (M_treshold)
**START CAMERA FEED:**   WHILE True: # Continuous video processing loop
      - Capture frame from the camera
      - Detect and track objects using fine-tuned model
      - Extract tracked objects (ID, class, bounding box)
      - Register detected objects
      **IDENTIFY PERSON–LUGGAGE INTERACTIONS:**
       
- For each detected luggage:
          
- Check if a tracked person is within R_own
          
- If a person is found:
- Associate the luggage with them          
- If no person is found:
- Start the abandonment timer    ** DETECT ABANDONED LUGGAGE:**
        
- If luggage is unassociated for more than T_threshold:
          
- Mark it as abandoned
          
- Trigger an alert (e.g., visual warning, alarm)
          
- Log alarm data


The system continuously monitors the location of the luggage and the IDs of the people to whom it is mapped, as well as other people entering the surveillance radius. If the luggage is in the same place for longer than the set limit, and all associated persons leave that radius, the system classifies the luggage as abandoned. Baggage is considered under supervision if, near it, there is at least one person in whose ownership it is. In addition, to reduce the number of false detections, the system compares the coordinates from multiple consecutive frames to filter out minor camera movements or vibrations and ignores luggage that is in motion, as it is assumed to be towed by the owner or transported by a vehicle or an automated conveyor belt.

C. Key Algorithm Parameters

**Figure 3:**
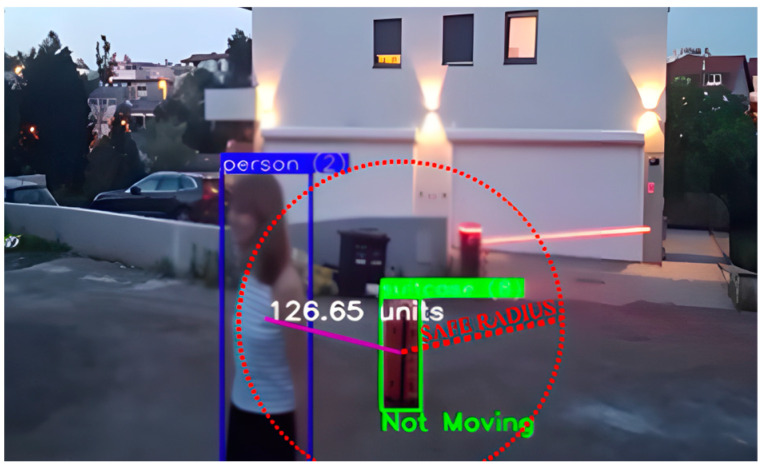
Person within safe radius of the suitcase. The image shows the detected person within the blue bounding box and the detected suitcase within the green bounding box, and the calculated distance between the center of gravity of the person and the suitcase. No movement of the suitcase was detected, so the label is “Not moving”, but the suitcase is not marked as abandoned because it is near the person within the safe radius marked by the red circle.

The key parameters in the algorithm for detecting abandoned luggage include the movement threshold, the radius of association (i.e., the distance between a person and the luggage) (Figure 3), and the duration the luggage remains stationary. These parameters were defined in advance based on the system configuration but can be adjusted to the specific characteristics of the environment and the technical recording conditions to improve the accuracy of abandoned luggage detection.

The movement threshold specifies the minimum displacement, measured in pixels, that luggage must undergo to be considered in motion. This effectively prevents false detections caused by noise or camera vibrations, while still reliably identifying actual movement. In practice, the luggage coordinates are stored for several previous frames, and if the difference in the luggage position exceeds the predefined threshold, the system concludes that the luggage is moving.

In this system, a luggage ownership radius of 40 px is suggested (height of average person detection), but this is a measure that can be adjusted for each system depending on the camera feed, as the size of objects varies depending on their distance from the camera. In this system, the time that was set for the luggage to be considered unattended was 10 s, but also this value is flexible and should be adjusted according to the system requirements.

Camera placement, angle, and distance from the scene also affect how the radius of association is determined, since perspective and a distant camera angle limit the level of detail, making the detected objects appear smaller and closer to each other compared to close-up footage. Consequently, these parameters must be adapted to real-world conditions to reduce false alarms and enhance the overall accuracy of the system. For example, in crowded spaces, a smaller radius of association can help avoid unnecessary links between luggage and random passersby. Conversely, a larger radius may be suitable for less crowded areas, where people typically stand further apart than in congested settings where they are forced closer together.

## 4. Development of Abandoned Luggage System

The development of the proposed system for detecting abandoned luggage comprised three key phases.

The first phase involved collecting and labeling images from real-world surveillance camera environments, thereby creating customized datasets for transfer learning across various public-space scenarios. The collected images were manually labeled to precisely identify objects such as luggage, persons, and other relevant objects, ensuring a reliable foundation for training the model.

The second phase centered on selecting an appropriate model architecture and training the object detection model, namely, fine-tuning it for detecting people and luggage captured by surveillance cameras, based on the customized datasets. It is essential that the model achieves high accuracy in detecting people and luggage, remains robust enough to perform well across diverse settings and under varying recording conditions, and is capable of real-time inference.

The third phase encompassed defining and implementing the algorithm for recognizing abandoned luggage.

## 5. Model for Person and Luggage Detection

In the context of abandoned luggage detection, it is crucial to achieve high precision and recall while operating in real time. Over the past several years, YOLO-family detectors have excelled in these areas, but the performance of visual transformers is also improving and achieving comparable results in object detection.

### 5.1. YOLOv8 and YOLOv11 Families

The YOLO architecture is based on a CNN that divides the input image into a grid of cells, with each cell simultaneously predicting bounding box coordinates, a confidence score, and object classes [14]. YOLO is a single-stage object detection method and has been in development since 2016, with two models being developed annually. One of the latest versions is YOLOv11 [7], and due to its excellent performance, YOLOv8 [7] is still very popular today. YOLOv8 was designed by the original YOLO team with a focus on improving the performance in the real-time detection of complex objects of varying sizes. YOLOv8 introduced a new dynamic prediction scheme that adjusts the number of model predictions according to the image complexity, resulting in reduced inference time and computing requirements. YOLOv8 includes several new optimizations, such as improved loss functions and better regulation, and a new pyramidal network architecture feature that improves the detection accuracy on small objects without compromising the detection accuracy on larger objects. YOLOv11 was developed in 2024 using the Ultralytics Python package and achieves higher accuracy with fewer parameters through innovations in model design and optimization techniques. The architectural innovations of the model include the introduction of the C3k2 (Cross-Stage Partial with kernel size 2) block, SPPF (Spatial Pyramid Pooling Fast), and C2PSA (Convolutional block with Parallel Spatial Attention) components, which contribute to the improvement of feature extraction [15].

Both YOLOv8 and YOLOv11 families of models come in different versions that are a tradeoff between computational complexity and model performance, from computationally lighter models (e.g., YOLOv8 Nano) to those with 20 times as many parameters (e.g., YOLOv8 XL).

In recent years, the goal of YOLO model development has been to extract and process features as efficiently as possible, while maintaining high accuracy [14,15]. The design of the YOLOv11 model has resulted in higher average precision (mAP) on datasets such as COCO, so that, for example, with 20% fewer parameters, it achieves even slightly better results than YOLOv8m. That is, with the same number of parameters as YOLOv8m, it achieves a 3% higher average precision, which roughly corresponds to the results achieved by the largest YOLOv8x model. A comparison of different versions of the YOLOv8 and YOLOv11 models is presented in Table 2. All models were pretrained on the COCO dataset, and more complex versions of the models of each family achieve higher accuracy but require more powerful computational resources [2].

The results that the YOLOv8 and YOLO11 models achieve on the COCO validation set potentially make them an excellent choice for our real-time detection scenario of surveillance camera footage and allow for their efficient implementation on resource-constrained devices, without compromising accuracy.

We tested all versions of the YOLOv8 and v11 models for inference speed on our computer (Table 2). The slowest were the x models, with a processing speed of about 36 ms, which is approximately 28 frames per second; so, they can be considered too slow or borderline acceptable for surveillance systems. All remaining YOLO models achieved inference speeds of less than 24 ms, which is approximately 42 frames per second. Since the human eye is considered to perceive motion as smooth at about 24 frames per second, 42 frames or more is an appropriate speed for surveillance systems. The performance of the models that met the requirements of our real-time detection system with the highest possible accuracy are marked in bold in the table (versions m and l of the YOLOv8 and YOLOv11 models).

### 5.2. DETR ResNet-50

The DEtection TRansformer (DETR) [16] with a ResNet-50 backbone is a transformer-based object detection model developed by Facebook AI. It has 41 million parameters and was pretrained on the COCO dataset. DETR replaces traditional object detection components with an end-to-end prediction approach. It utilizes a bipartite matching loss for direct object detection without post-processing. The model efficiently captures long-range dependencies and contextual relationships in images, which made it potentially interesting for our detection task.

Table 2 shows that the DETR model has a similar number of parameters as the YOLOv11x model or YOLOv8l but achieved 8% less accuracy and required 93.6 ms per frame for inference, that is 10 FPS, which makes it unsuitable for real-time inference.

We also considered the model’s robustness considering the need for domain shift [2], a situation where the distribution of the training data differs from that of the real-world environment. In our case, our models were trained on the COCO dataset but are intended for airport scenes recorded by surveillance cameras from a bird’s-eye view. To mitigate the effects of domain shift and varying recording conditions and to adapt the model to the target domain, we employed transfer learning, which simplifies the transition from one image type to another while reducing both the training time and the need for a large volume of manually annotated samples [2,3,10].

Considering previous experiences with tuning the YOLOv8 model for images captured from a bird’s-eye view [17,18] as well as the performance that the models achieve in real-time, we selected the YOLOv8m and YOLOv11m models for further testing on domain shift of object detection in surveillance camera images.

## 6. Custom CCTV Datasets of Public Places

In order to adapt the detection model to the specific conditions of recording in open and closed public spaces and the perspective of surveillance cameras, two customized datasets were created from two locations, using surveillance camera footage from a pedestrian zone in the center of Rijeka, Croatia, called Korzo, and footage from a surveillance camera at Düsseldorf Airport, Germany. Both cameras record 24 h a day throughout the year; so, short videos recorded over several months at different times of the day were sampled to capture different recording conditions with respect to lighting and changes in the number of people in the scenes.

The frames were sampled from the videos, so that one frame was taken every second, which was experimentally determined to be a sufficient frequency to detect changes in a scene related to the presence of luggage and people’s behavior. This sampling method was consistently applied to the recorded material, and three customized datasets were defined, as follows: CCTV-Korzo, containing images from the city promenade, CCTV-Düsseldorf from the airport, and their combination CCTV-KD.

### 6.1. CCTV-Korzo Dataset

**Figure 4:**
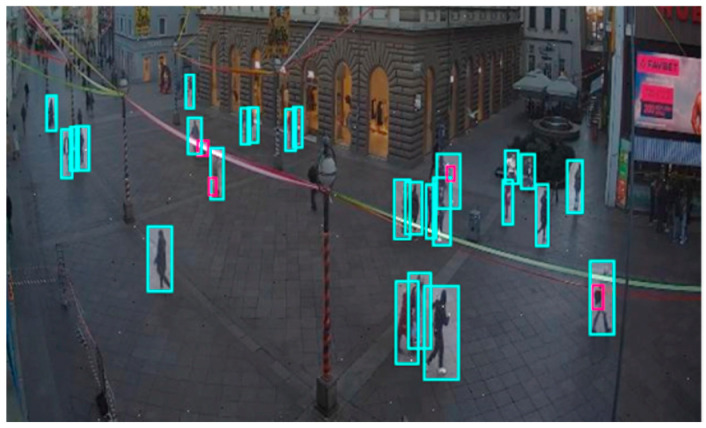
Image from the CCTV-Korzo dataset with annotated people marked with a light blue bounding box.

This dataset contains images of outdoor scenes captured by a surveillance camera on the pedestrian zone Korzo, the city’s main promenade in Rijeka, featuring people in motion (Figure 4). The CCTV-Korzo dataset comprises 60 images. The images had an average size of 942 × 526 pixels and were resized to 640 × 640 pixels. Augmentation processes were made such as horizontal flipping, saturation shifts between −25% and +25%, exposure adjustments between −10% and +10%, and adding noise up to 0.1% of pixels; with augmentation, the dataset was expanded to 100 images. The Korzo dataset contains 1423 annotated objects, of which 1299 refer to people, and only 124 to luggage.

Although the CCTV-Korzo dataset has a relatively small number of luggage annotations (around 8% of the total annotations), it is valuable because it contains perspectives that closely resemble footage from surveillance cameras in public spaces.

### 6.2. CCTV-Düsseldorf Dataset

**Figure 5:**
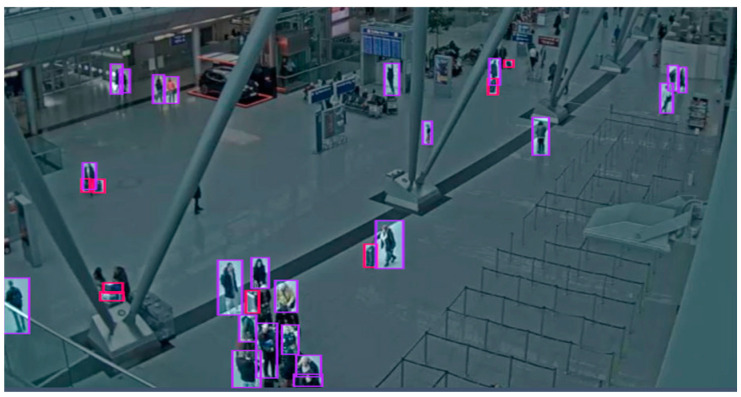
Labeled image from CCTV- Düsseldorf dataset so that people are marked with a purple bounding box and suitcases with a red.

The **CCTV-Düsseldorf** dataset includes images captured indoors at different times of the day and under various lighting conditions (Figure 5). It consists of 240 images with a median image ratio of 1913 × 954 pixels. The images were resized to 640 × 640, and augmentation processes such as horizontal flipping, rotation between −13° and +13°, hue shifts between −21° and +21°, and exposure adjustments between −5% and +5% were applied to the training set. In this set, fewer augmentation image transformations were used than in the Korzo set, because the set already contains different characteristic scenes in the closed space of the airport and a larger number of luggage objects. After augmentation, our dataset has 350 images, with 7751 object annotations, namely, 5115 person annotations and 2636 luggage annotations.

### 6.3. CCTV-KD Dataset

The dataset that includes images from the Korzo and Dusseldorf sets is called the CCTV-KD set. It consists of 474 tagged images with an average resolution of 1692 × 614 pixels and includes a total of 9174 object annotations, including 6414 person tags and 2760 luggage tags.

**Figure 6:**
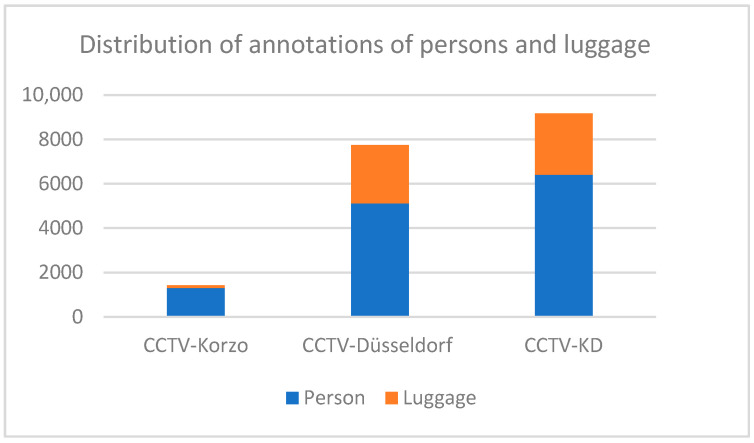
Distribution of annotations of persons and luggage in surveillance sets CCTV-Korzo, CCTV-Dusseldorf, and CCTV-KD.

**Figure 7:**
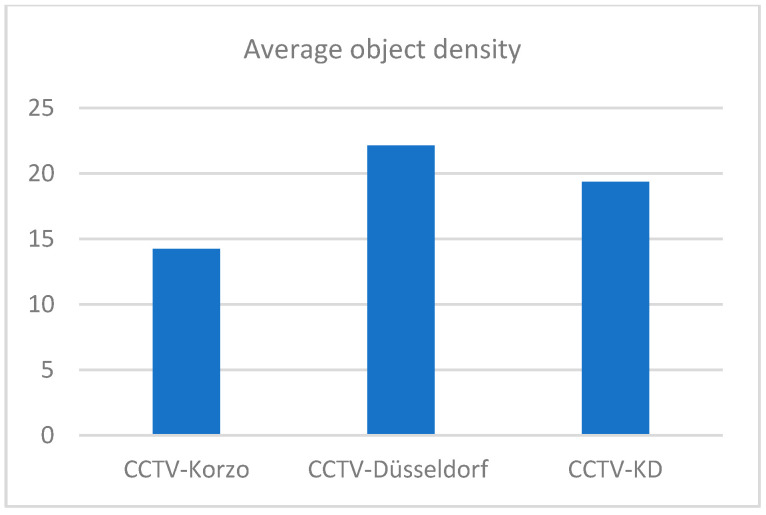
Average object density in custom sets CCTV-Korzo, CCTV-Dusseldorf, and CCTV-KD.

The distribution of the annotations of people and luggage in our surveillance datasets CCTV-Korzo, CCTV-Dusseldorf, and CCTV-KD is shown in Figure 6. The images have an average of 20 annotated objects of interest (from 14 in CCTV-Korzo to 22 in CCTV-Dusseldorf), given that the scenes were filmed at different times of the day when the number of people in them changed significantly, and only those objects that were large enough (more than 10 px) to be discerned and that were not obscured by large occlusions were marked. The average density of the objects in the clusters is shown in Figure 7.

## 7. Experiment Setup

### 7.1. Evaluation Metrics

In order to select the best performing model for person and luggage detection, standard metrics were used to quantitatively assess the detection accuracy of the model such as precision, recall, and mean average precision (mAP) [14], with a minimum confidence threshold of 50%. Precision and recall evaluate the quality of a model: precision is the ratio of correct predictions to the total number of predictions, measuring a model’s ability to identify only relevant cases; recall is the ratio of true positives to all possible positives in a dataset, measuring a model’s ability to find all relevant instances.

**Figure 8:**
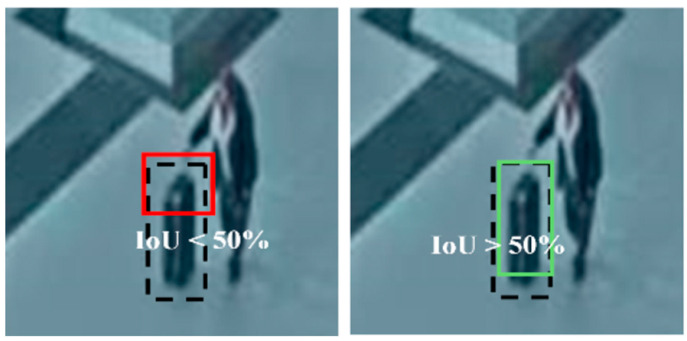
A detection that has an intersection over union with ground true greater than or equal to 50% is considered good and is marked with a green box in the image, and one that is less than that is considered bad and is marked with a red bounding box.

Additional metrics specific to object detection include mAP@0.5. The mAP@0.5 metric measures detection accuracy at a 50% overlap threshold between the predicted bounding box and the ground truth box (Intersection over Union, IoU). For detecting abandoned luggage in our scenario, pinpoint bounding-box accuracy was not strictly necessary, making the 50% IoU (Figure 8) accuracy threshold the most relevant value.

### 7.2. Training Setup

The original versions of the YOLOv8m, YOLOv11m, and DETR (Resnet-50) models were fine-tuned on the prepared datasets CCTV-Korzo (K), CCTV-Düsseldorf (D), and combined CCTV-KD (KD), and then all models were tested on the test set collected at Dusseldorf airport that none of the models had seen before. The CCTV-Korzo, CCTV-KD, and CCTV-KD-E datasets were split in two parts, i.e., 80% for training, and 20% for validation, because all tests were performed on the Dusseldorf test set. The CCTV-Dusseldorf set was therefore split in three parts, corresponding to 75%, 10%, and 15%, respectively, for training, validation, and test. We also evaluated the results against those of the original versions of the models to assess the extent to which fine-tuning and domain optimization contributed to domain adaptation and performance enhancement.

For all models, we used the standard configuration and trained them for two classes (person and luggage), with a composite loss and the SiLU activation function, a batch size of 16, and momentum of 0.937. The loss function was YOLOv8’s default composite loss, combining binary cross entropy for classification, distribution focal loss, and CIoU loss for bounding box predictions, and an IoU-aware classification score. The input images were resized to 640 × 640 pixels during data augmentation by directly stretching them to the target resolution, preserving consistency across the dataset for optimal training and detection. Each model was trained for up to 150 epochs or until the loss ceased to decrease.

**Figure 9:**
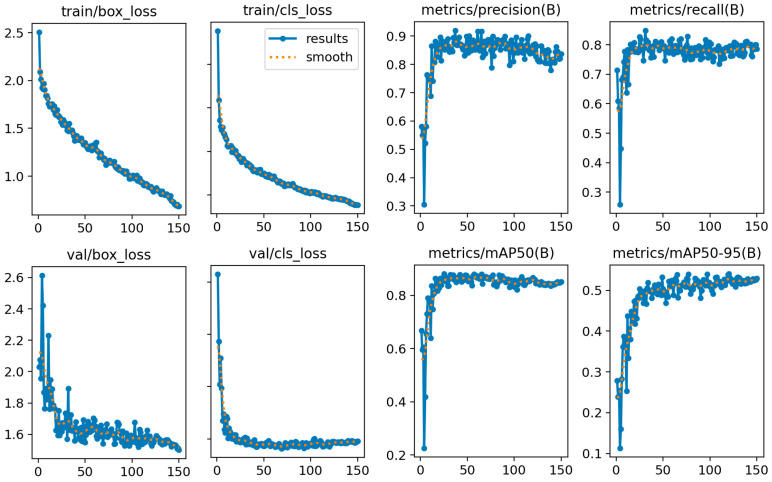
Training results for YOLOv8-m.

Using the example of the YOLOv8-m model, Figure 9 shows the performance metrics used during model fine-tuning. The model consistently reduced the error to below 0.5 over 100 epochs, indicating that the model was effectively learning to classify objects in the training set and that it achieved convergence in the validation set after 20 epochs, indicating good generalization to unseen validation classes. The precision and recall at the bounding box level quickly stabilized and reached high values of 0.8–0.9, demonstrating the model’s ability to correctly identify positive detections and to find real objects in the images. The model achieved high detection accuracy at the standard evaluation threshold of 50% IoU and typically slightly lower performance at more strict evaluations over higher IoU thresholds of 50–95%. All graphs demonstrated good convergence and stability of the metrics and successful fine-tuning of the model. During training, YOLO automatically evaluated the validation performance at each epoch and saved the best-performing model checkpoint as best.pt. This selection was based on the lowest validation loss and overall metric improvements. Accordingly, for evaluation and testing, we used the best.pt model for all versions of fine-tuned models in our experiments.

## 8. Evaluation of the Fine-Tuned Person and Luggage Detection Models

### 8.1. Quantitative Comparison of Models Trained on Different Domain Sets

**Figure 10:**
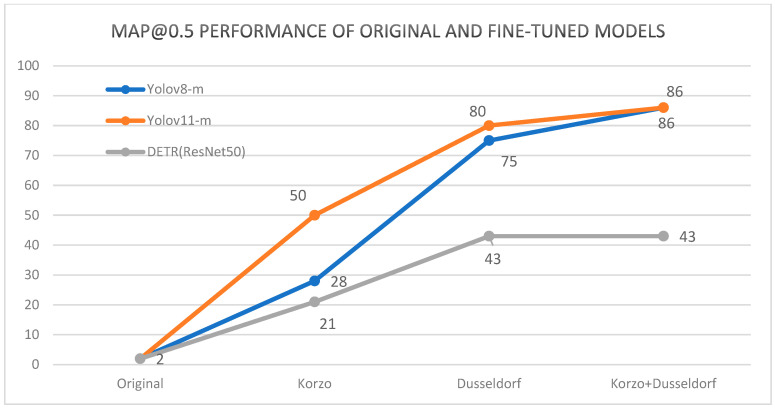
Performance comparison of the original models and the models fine-tuned on the different K, D, and KD sets and tested on the Düsseldorf test set.

The results of the models that were domain-optimized on the CCTV-Korzo, CCTV-Dusseldorf, and CCTV-KD sets for the Dusseldorf test set according to the mAP@0.5 metric are shown in Figure 10. The graphs show that the original results for all models improved significantly after fine-tuning for dataset optimization. For instance, the YOLOv8m model trained on CCTV-KD achieved an mAP@0.5 of 86.44%, whereas the baseline model only achieved a mAP of 3.34%. Both YOLO models achieved the best results on the combined KD set, even 5% better than on the CCTV-Düsseldorf set, while the DETR model achieved equal cuts on both sets. It is interesting to note that the KD set is an extension of the Düsseldorf set, with the images of surveillance cameras on the Korzo city promenade, which shows that the inclusion of images taken from a similar perspective from another domain had a positive effect on improving the performance of the models. Interestingly, both YOLO models fine-tuned on the KD set achieved even 40% better results than the fine-tuned DETR model. The significantly worse results of the DETR transformer compared to the YOLO models were probably due to small-size objects in the images that the model failed to detect, since previous studies have shown that transformers often struggle with detecting small objects due to their global attention mechanism, as they can dilute fine-grained details [12].

### 8.2. Analysis of the Impact of the Training Set Size on Model Performance

The scenes we used to train the model were crowded with objects, and the question arose as to how increasing the number of images in the set affects the performance of the model. On average, there were 20 objects of interest in the images; so, an increase of 100 images resulted in an increase of 1500 to 3000 objects.

We tested the YOLOv8-m model on three variants of the set in which we took a random 65% of the images from the CCTV-KD set, the CCTV-KD set, and the expanded version of the CCTV-KD set. The expanded KD version includes additional images from the Düsseldorf surveillance camera footage, so that it has a total of 570 images and 11,104 object annotations, of which 7890 person tags and 3214 baggage tags. The added images underwent the same preprocessing and frame selection process from the video as the images in the original dataset.

The performance of the YOLOv8-m model optimized on different training set sizes is shown in Table 3. The model did not show significant improvements when trained on a larger dataset (CCTV-KD-E); however, reducing the dataset by 35% (CCTV-KD-65%) resulted in a performance degradation in all metrics of about 7%. Accordingly, the original CCTV-KD dataset was selected in further experiments for model fine-tuning as the best tradeoff between model performance and dataset complexity.

### 8.3. Quantitative Results Related to Model Size and Discussion

The performance of the YOLOv8 and YOLOv11 models, fine-tuned on the KD dataset (labeled as YOLOv8(KD) and YOLOv11(KD)), was analyzed in detail, as these models delivered the best detection results. To further evaluate their effectiveness, all versions of the models (nano, small, medium, and large) were fine-tuned on the same KD dataset. Table 4 compares the performance of the YOLOv8(KD) and YOLOv11(KD) model variants across different sizes using three key metrics: precision, recall, and mAP@50. The results were achieved on the validation set during model training and showed the performance achieved in the best epoch of model learning.

All versions of the YOLOv8(KD) and YOLOv11(KD) models demonstrated strong and consistent performance across all key metrics. Precision ranged from 84.1% to 91.2%, with the highest value achieved by the YOLOv11(KD)-s model. Recall varied from 77.7% to 80.3%, with the best result observed for YOLOv8(KD)-n. In terms of mAP@50, the scores ranged from 85.1% to 88.3%, again with the YOLOv11(KD)-s model performing best.

Across most equivalent model variants (e.g., when comparing nano to nano, medium to medium versions), the performance differences between YOLOv8 and YOLOv11 were generally minor, typically within ±2%. However, a notable exception was the small model, where YOLOv11(KD)-s outperformed YOLOv8(KD)-s by 7% in precision.

Overall, the difference in performance between YOLOv8(KD) and YOLOv11(KD) was relatively small. The choice between the two will depend on the specific requirements of the application; in our case of abandoned luggage, it is more critical that the model achieves higher recall in order to detect as many potentially suspicious objects as possible and to provide our abandoned luggage detection algorithm with the highest quality input data.

**Figure 11:**
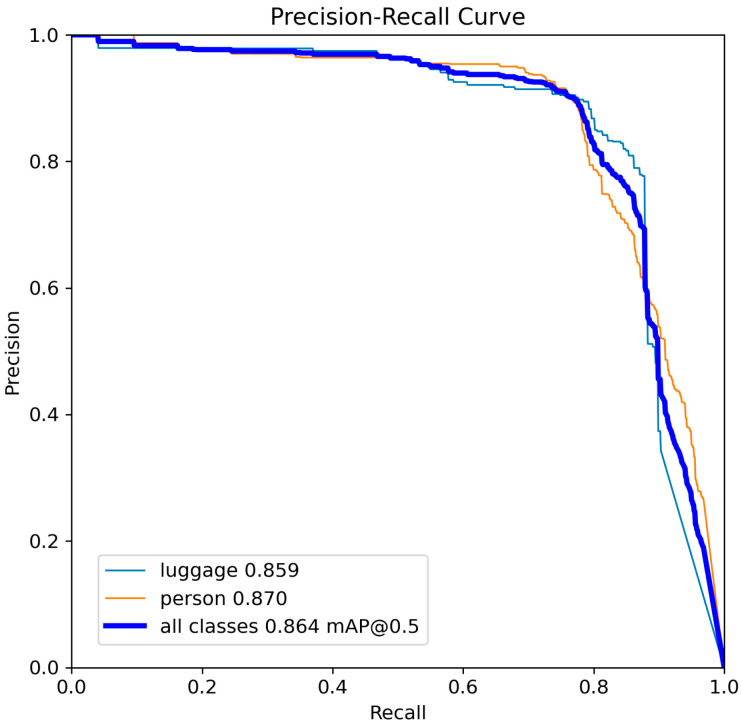
Precision-recall curve for YOLOv8-s.

**Figure 12:**
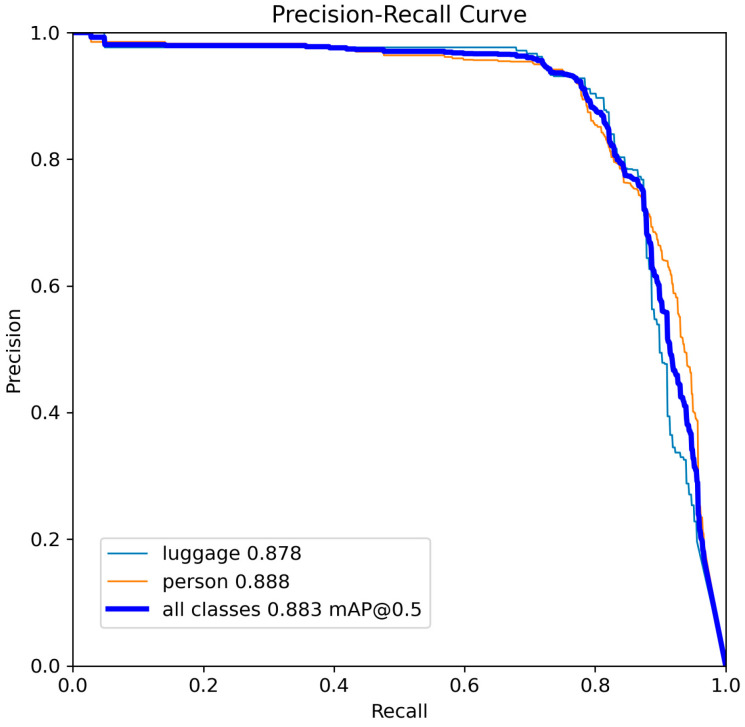
Precision-recall curve for YOLOv11-s.

For the best optimized models YOLOv8-s and YOLOv11-s, the precision–recall curve is shown, showing their relationship with respect to the standard Intersection over Union (IoU) threshold of 50% and the selected model confidence level (Figure 11 and Figure 12).

### 8.4. Quantitative Results Related to Object Size and Discussion

Given the similar properties and performance of the YOLOv8(KD) and YOLOv11(KD) models, we tested their performance in relation to the size of the objects to be detected on the Düsseldorf testing dataset. Since, in our case, we were dealing with surveillance camera footage, we mainly had medium-sized objects and many small objects in the scenes; so, the important information for model selection, in addition to real-time performance, was how the model performed in detecting medium-sized and small objects. For this purpose, we tested the optimized YOLOv8 and YOLOv11 models considering the COCO detection evaluation metric.

Per the COCO standard, object sizes are categorized as follows:

- Small objects: area < 32^2^ pixels.
- Medium objects: 32^2^ ≤ area < 96^2^ pixels.
- Large objects: area ≥ 96^2^ pixels.

**Figure 13:**
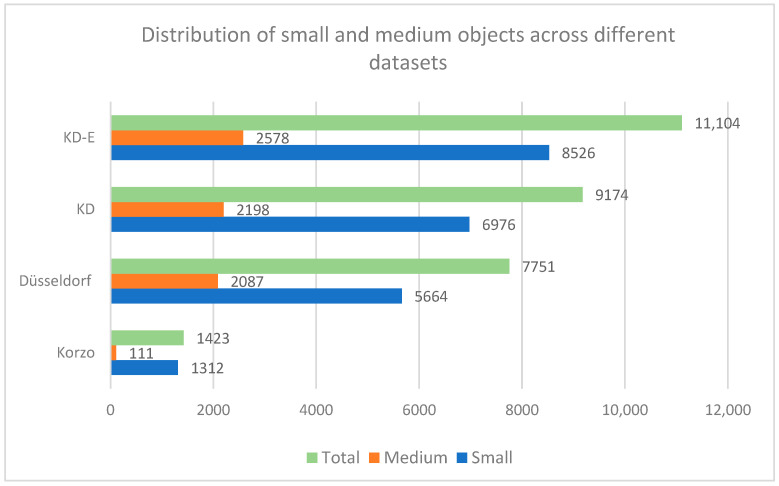
Distribution of small and medium objects across different datasets.

The CCTV-KD dataset included no large objects, resulting in an AP_large score of −1.0, primarily due to the security camera setups covering wide areas, causing objects to appear smaller and further away. In total, the dataset contained 9174 labeled objects, of which 6976 small objects and 2198 medium objects (Figure 13), confirming the absence of large objects.

**Figure 14:**
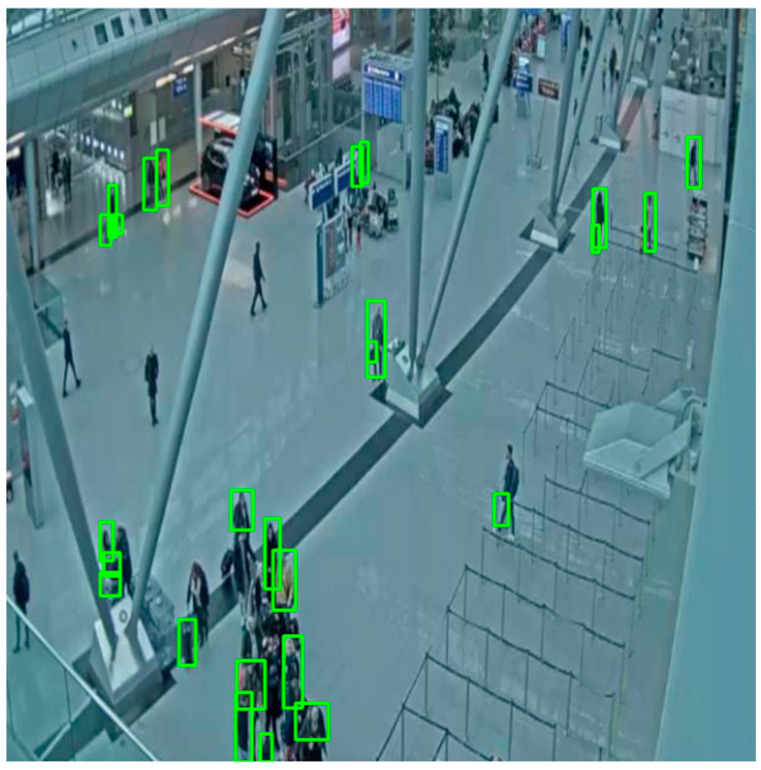
Small-size objects detected with Yolov8(KD)-m and marked with green bounding box.

**Figure 15:**
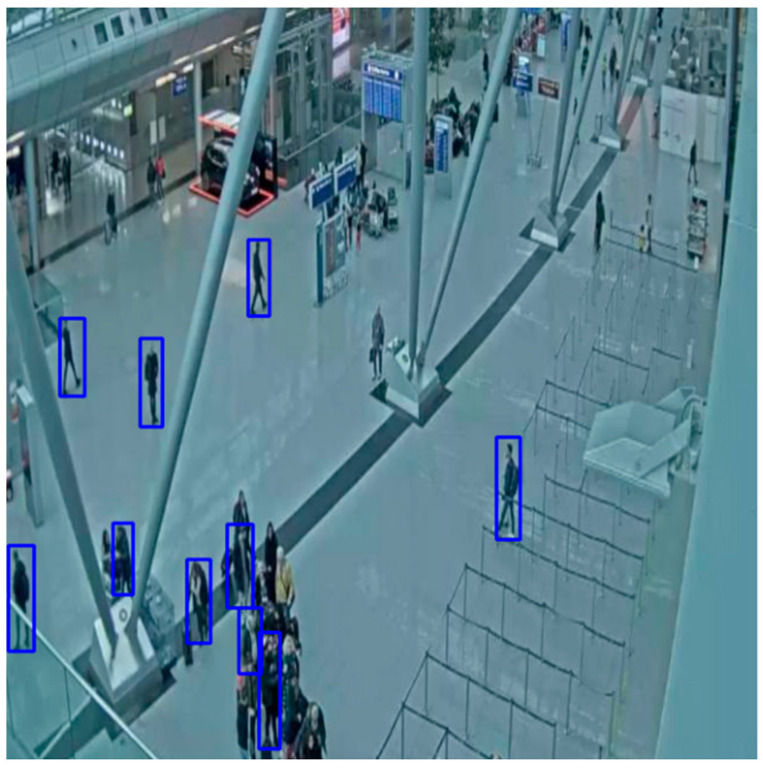
Medium-size objects detected with Yolov8(KD)-m and marked with blue bounding box.

Examples of small objects in our CCTV-KD dataset are compact bags, backpacks, or people who were further away from the surveillance camera, as shown in Figure 14; medium-sized objects are shown in Figure 15. The detection of small objects was challenging for the models, due to insufficient spatial and semantic information at deeper network feature layers, which is crucial for accurate detection.

The performance of the fine-tuned YOLOv8(KD) and YOLOv11(KD) models from their small (s) to their large (l) versions, with a particular focus on their ability to detect small and medium-sized objects, is shown in Table 5.

The results showed that YOLOv8(KD) and YOLOv11(KD) performed similarly for small and medium object detection and achieved an mAP@0.5 in the range from 86 to 88%. For small object detection, YOLOv11(KD)-s showed a slight improvement, with an AP_small of 85.8%, while the second best performer was the YOLOv8-l model, with an AP_small of 84.5%, a result almost aligned with those achieved by the small YOLOv8 model and the YOLOv11 l version. All models performed well in detecting medium-sized objects, with YOLOv8(KD)-l achieving the highest AP_medium of 96.4%, followed by YOLOv8(KD)-s, with an AP_medium of 94.7%, and YOLOv11(KD)-s slightly lagging behind, with an AP_medium of 93.4%

Overall, the YOLOv11(KD)-s and YOLOv8(KD)-l models achieved equally good results, with equally high values of mAP@0.5 precision, but the s version of the YOLOv11 model obtained better results in detecting small objects, and the l version of YOLOv8 was better in detecting medium-sized objects. In our case, it is important that the model achieves better results in the detection of small objects, because there are many more of them in the scenes of interest, and that it allows for fast detection; so, we chose the YOLOv11(KD)-s model as part of our abandoned luggage detection system.

### 8.5. Qualitative Comparison

**Figure 16:**
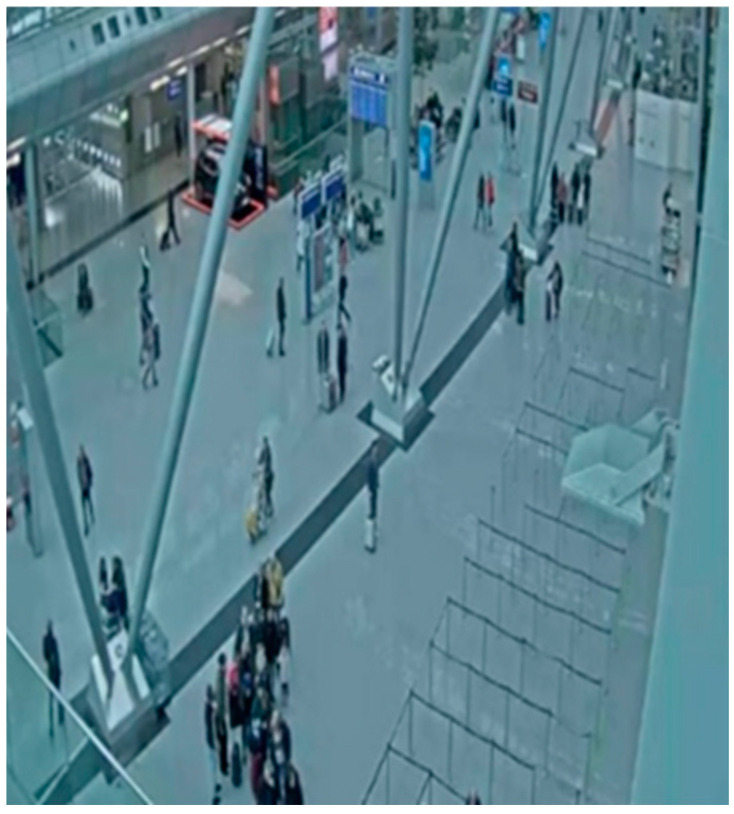
YOLOv8-l baseline model who did not detect any people or luggage at the scene.

**Figure 17:**
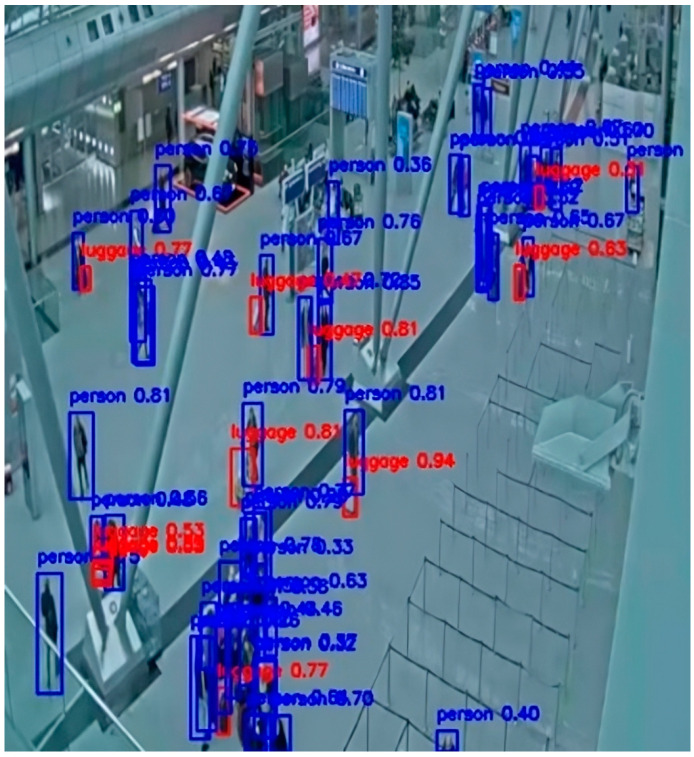
YOLOv8(KD)-l fine-tuned model with on-site person and luggage detections. Detected persons are marked with a blue border box and luggage with a red one, and a confidence score is attached to each one.

**Figure 18:**
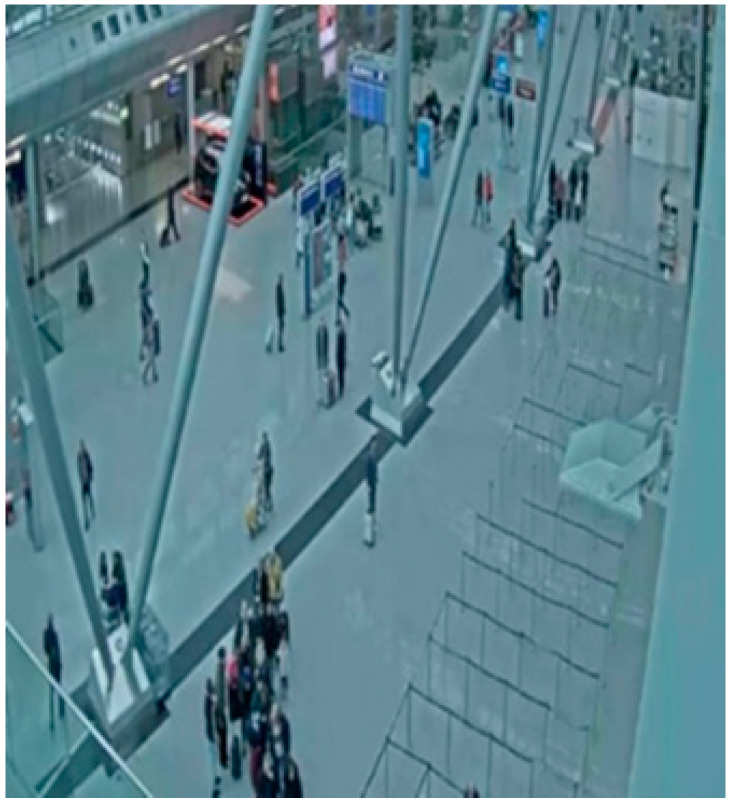
YOLOv11-s baseline model who did not detect any people or luggage at the scene.

**Figure 19:**
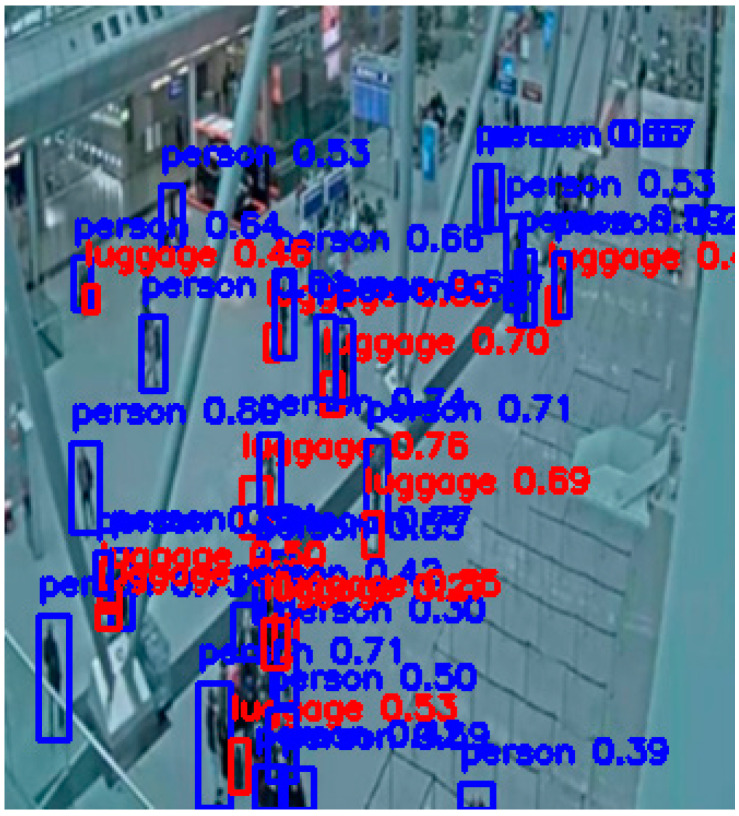
YOLOv11(KD)-s fine-tuned model with on-site person (blue bounding box) and luggage (red bounding box) detections along with confidence score.

Below are examples of detections by the basic YOLOv8-l and YOLOv11-s models and their fine-tuned versions. The baseline models of both versions failed to detect either people or luggage (Figure 11 and Figure 13), clearly highlighting their limitations in real-world surveillance scenarios. In contrast, after the models were domain-optimized on the CCTV-KD set, they successfully detected both people and luggage (Figure 12 and Figure 14). This difference highlights the importance of fine-tuning a model for specific application domains such as person and baggage detection, in order to effectively assist surveillance operators in protecting areas against potential security risks (see Figure 16, Figure 17, Figure 18 and Figure 19).

## 9. Different Scenarios for Testing the Abandoned Luggage Detection System

Detecting abandoned luggage in a real-world environment involves addressing different scenarios that reflect different human behaviors and interactions in public spaces. In the following, we elaborate and analyze the potential scenarios that we considered in this research to test the proposed system based on our fine-tuned YOLOv11(KD)-l model for detecting and tracking people and luggage. Each real-world scenario presents specific challenges; so, the parameters need to be adaptable, and the abandoned luggage detection algorithm must be robust.

### 9.1. Single Person Abandoning Luggage

**Figure 20:**
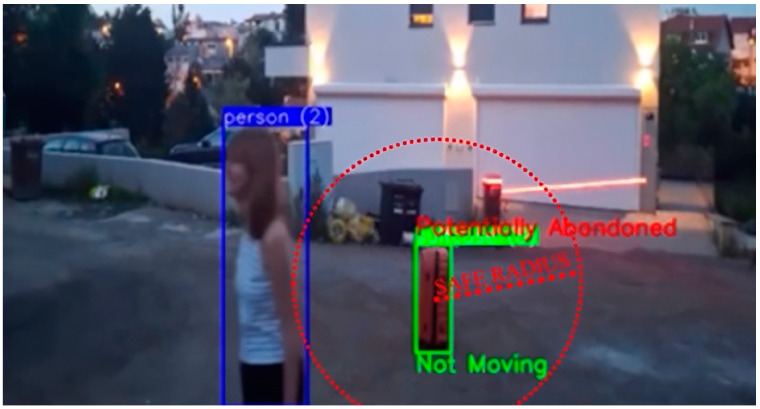
Single person abandoning luggage. The image shows the detected person within the blue bounding box and the detected suitcase within the green bounding box, and the safe radius marked by the red circle around the suitcase that is no moving so the associated label is “Not moving”. Person is leaving the safety radius of the luggage, so the luggage is marked as abandoned.

The simplest scenario involved a single person entering the monitored area with one or more pieces of luggage and subsequently leaving the area, leaving the luggage unattended. The system must accurately associate the person with their luggage, track their movements, and confirm that the individual has exited the predefined radius while the luggage remains stationary for a specified duration (Figure 15). Our system worked without any problems in this test scenario (see Figure 20).

### 9.2. Group of People with Luggage

In this scenario, a group enters the monitored area, potentially with multiple pieces of luggage. Some members of the group may leave the area while others remain, maintaining supervision over the luggage. Importantly, the system must distinguish between separate individuals or groups simply passing by each other and cohesive groups, such as families who share luggage. Misidentifying these relationships can lead to false abandonment alarms. To address this, the system employs ID-based tracking to associate each piece of luggage with the correct individual(s), allowing for the accurate mapping of luggage ownership within dynamic group interactions. After initial detection and tracking, an algorithm continuously evaluates the positional relationships and assigned IDs to update the group memberships and maintain consistent ownership links.

Our system is designed for these situations and did not raise false alarms when ownership was transferred from an individual to a cohesive group.

### 9.3. Restroom Break

A common real-world scenario occurs when multiple individuals share a single piece of luggage but take turns leaving the immediate area, such as when using the restroom. For example, two travelers arrive with a single suitcase, and Person #1 briefly goes to the restroom, while Person #2 stays with the luggage; then they switch, and Person #2 goes to the restroom, while Person #1 guards the luggage. The system detects the event that the original “owner” has left the safe baggage-detection radius, but if another member of the group is within the set radius, the baggage is never marked as unattended.

This scenario highlights the importance of the robust modeling of group ownership and flexibly associating baggage with owners. Our system allows for the transfer of ownership from Person #1 to Person #2 (and vice versa), thereby preventing false alarms resulting from consecutive, short-term abandonments of the baggage. Our solution involves the introduction of a proximity-based timer, whereby an individual who remains near a piece of baggage for a sufficient period is dynamically remapped as its active owner. Additionally, each time the baggage begins to move—such as when lifted or rolling—the system can transfer ownership to the person physically handling it, thereby reducing false alarms associated with short, consecutive departures.

### 9.4. Shaking and Video Disruptions

Shaking and video disruptions, often caused by unstable camera setups or environmental factors (e.g., intense vibrations during airplane takeoff), create blurred frames, motion distortions, and inconsistencies in object trajectories, reducing the reliability of standard detection and association algorithms. To counteract these effects, our system applies an averaging mechanism over a variable number of recent frames (determined by camera specifications and environmental conditions), combined with tailored movement thresholds, ensuring that transient vibrations and brief motion anomalies are sufficiently smoothed out to prevent false detection or missed associations.

### 9.5. Crowded Environments

**Figure 21:**
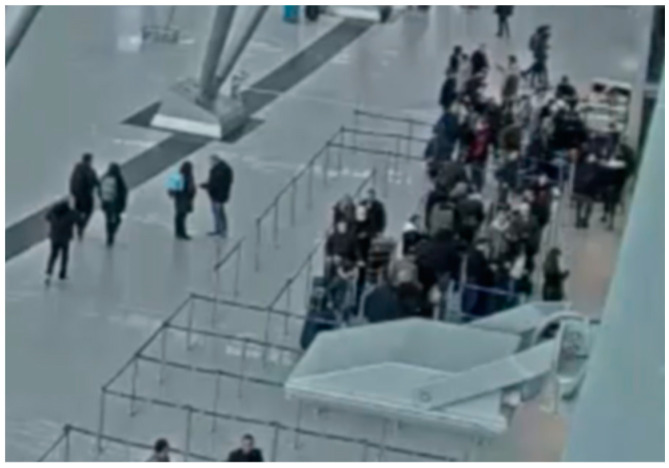
Crowd at the airport, people are standing in line for baggage claim.

Crowded settings, such as airport terminals or public plazas, introduce significant challenges for abandoned luggage detection. Large numbers of people and objects move through a scene, frequently overlapping and covering each other. This results in luggage sometimes being partially or completely hidden from the camera’s view, complicating its continuous tracking and luggage association with the owner. In addition, multiple similar-looking items may be present, creating ambiguity when trying to maintain the identity of each piece of luggage (Figure 21).

Detecting abandoned luggage in densely populated areas is inherently challenging and often impractical, as occlusions and constant motion make it nearly impossible to reliably identify stationary objects.

Therefore, crowded scenarios are usually not specific to the task of detecting abandoned luggage, although they are crucial for increasing public safety in large traffic hubs; so, we analyzed them as well. Also, today’s detectors, including our system, have problems detecting people and luggage in these cases, especially caused by changes in the identity of the luggage owner due to temporary obscuration from the camera and occlusions, which also affects the ability to determine the belongings of the luggage.

To address these challenges, our system uses adaptive parameters such as luggage ownership radius, movement threshold, and inactivity duration; however, small objects and the problem of interruptions in person and luggage tracking due to occlusion and overlap are obstacles that cannot currently be successfully overcome.

## 10. Discussion

The assumptions and adjustable parameters incorporated into our algorithm demonstrated sufficient flexibility to enable the detection of abandoned luggage across diverse real-world scenarios. Despite these strengths, the system exhibits notable limitations in densely crowded environments, primarily due to the constraints of the object detector. Challenges arise when detecting small objects positioned far from the camera, as well as in instances of object overlapping, occlusion, or high crowd density. These limitations are not unique to our system; even human annotators frequently encounter difficulties in such conditions, occasionally overlooking partially occluded objects and thereby producing incomplete ground-truth annotations. This can result in scenarios where correctly detected objects are misclassified as false positives due to incomplete labeling.

For potential edge-based deployments, knowledge distillation techniques that transfer information from larger teacher models to smaller student models could help optimize inference on resource-constrained devices [15]. However, the YOLO models, particularly the small versions known for their computational efficiency and lightweight architecture, are inherently well-suited for edge devices without requiring additional student models for optimization, unlike transformer-based models that often demand significant resources. In centralized surveillance systems, video feeds are typically processed at a central station, where computational efficiency is less of a constraint. In such cases, prioritizing robust processing capabilities to handle high-resolution video streams and complex analytics is more beneficial than focusing on model compression for edge deployment.

Earlier research [1,2,4] demonstrated higher detection accuracy, but this success can be attributed to the simpler conditions under which their systems were evaluated. These studies predominantly focused on detecting single, isolated, abandoned objects in controlled or less dynamic environments, where the absence of complex crowd interactions and occlusions facilitated a better performance. Although our system has not yet reached the detection accuracy observed in such controlled conditions, it leverages advancements in modern object detection frameworks to address more challenging, real-world environments in real time.

Future improvements in luggage detection systems will focus on enhancing performance in crowded environments by integrating advanced techniques like inter-frame multiscale probabilistic cross-attention mechanisms [19] into YOLO-based models. By utilizing spatial–temporal information from consecutive video frames, these systems can better detect small, occluded, or motion-blurred luggage items while minimizing background noise. Additional advancements, such as multi-camera surveillance setups, improved occlusion-handling algorithms, and robust re-identification methods, will further enhance the ability to track luggage and its owners in dense pedestrian areas with frequent obstructions. These innovations are essential for developing reliable abandoned luggage detection systems capable of operating effectively in real-world, high-traffic scenarios, ensuring both security and efficiency.

## 11. Conclusions

This paper presented a prototype system for abandoned luggage detection that can shorten the response time of surveillance teams to suspicious situations, thereby reducing the need for manual monitoring and contributing to a higher level of safety in public spaces. The research aimed to develop a model that automatically recognizes abandoned luggage in surveillance footage, detects situations where luggage has been left unattended, and alerts the appropriate services.

For this purpose, data were collected from surveillance cameras in public places such as a city promenade and an airport to form sets of surveillance scenes for training and testing the model. The deep convolutional models YOLOv8, YOLOv11, and transformer DETR (ResNet-50) were selected for detection, since they have achieved good detection results on the COCO data set, and were subsequently fine-tuned on our custom set. All original models produced extremely poor initial results; however, they showed significant improvement after fine-tuning, e.g., the YOLOv8-m model mAP@0.5 rose from only 3% to 86%, and the YOLOv11 model behaved similarly. The DETR model failed to adjust to the same extent on our custom set and was significantly slower; so, we dropped it as a solution.

Additionally, considering that surveillance scenes abound with objects that are small or medium in size according to the COCO evaluation metric, we additionally fine-tuned all the variants of the YOLOv8 and YOLOv11 model families. All models showed excellent performance in detecting medium-sized objects, with accuracy of up to 96.4% achieved by the large version of the YOLOv8 model. The performance of the models in the detection of small objects was somewhat lower, but still very good, corresponding to 85.8% for the YOLOv11-s model, which also showed the best mAP@0.5 of 88%; so, we chose it as part of the luggage detection system

The experiment showed that by adapting the model to the dataset, it will provide significantly better results that are crucial for further analysis and use in the proposed algorithm for automatic detection of abandoned luggage. Our algorithm is defined so that it classifies detected luggage as abandoned if it is stationary for more than a defined time threshold and if it is out of reach of all owners within a defined radius. Different scenarios of abandoned luggage in different environments were analyzed, including city streets, promenades, and airports, and the model was tested on footage of different scenarios in order to more accurately assess its performance and limitations in different security contexts.

The study confirmed that the fine-tuned YOLOv11-s model can be effectively adapted to a specific dataset and successfully employed for detecting abandoned objects in real-world conditions. Future work will focus on analyzing various scenarios of luggage abandonment and optimizing the algorithm parameters, particularly regarding different recording conditions, to further tailor the system to high-risk situations.

## Figures and Tables

**Table 1:** Comparison of different approaches for abandoned luggage detection.

Approach	Refs.	Main Features	Advantages	Disadvantages
Background Subtraction Methods (traditional)	[1,3,4]	Static/moving parts of the image—Foreground segmentation	Simple to implement—Low computational requirements	Sensitive to crowded scenes—Low accuracy
MCMC and SVM	[2,3]	Bayesian networks—Classification (SVM)	Tracks short displacements well	Sensitive to variations
Hybrid Approaches (CNN + Background Subtraction)	[1,5,7]	Combination of background subtraction and CNN	Improved accuracy—Fewer false positives	Limited to sudden changes
YOLO	[4,7,8,9,10,11]	Single-pass detection—Transfer learning	High accuracy even in crowded scene—Easily adaptable	GPU-intensive during training

**Table 2:** Comparison of YOLOv8, YOLOv11, and DETR (ResNet50) original models. The most suitable models for our task are highlighted in bold.

**Model**	**YOLOv8**				**YOLOv11**			
**Version**	**Number of Parameters (M)**	**Model Size (MB)**	Inference Time (ms) *	**mAPval** **(0.50:0.95)**	**Number of Parameters (M)**	**Model Size (MB)**	Inference Time (ms) *	**mAPval** **(0.50:0.95)**
Nano—n	3.2	8.9	11.04	37.3	2.6	9.9	10.90	39.5
Small—s	11.0	30.6	9.86	44.9	9.4	35.8	10.85	47
Medium—m	**25.0**	**68.9**	**16.71**	50.2	**20.1**	**76.7**	**16.75**	51.5
Large—l	40.0	113.3	23.98	**52.9**	**25.3**	**96.5**	**20.39**	**53.4**
XL—x	68.0	189.0	36.48	53.9	56.9	217.1	36.30	54.7
**Model**	**DETR**							
**Version**	**Number of Parameters (M)**	**Model Size (MB)**	Inference Time (ms) *	**mAPval** **(0.50:0.95)**				
ResNet-50	41.0	156.4	93.6	45.9				

* Tested on NVIDIA (NVIDIA Corporation, Santa Clara, CA, USA) GeForce RTX 2080 T, driver version: 550.107.02, CUDA version: 12.4.

**Table 3:** Comparison of YOLOv8-m results on KD datasets of different sizes. The best results are highlighted in bold.

Model	mAP@50	mAP@50-95	Precision	Recall
CCTV—KD-65%	0.7918	0.3729	0.8264	0.7013
CCTV—KD	0.8644	**0.5424**	**0.8943**	**0.7768**
CCTV—KD-E	**0.8771**	0.5318	0.8916	0.7725

**Table 4:** Comparison of the results of the YOLOv8(KD) and YOLOv11(KD) family models. The best results are highlighted in bold.

Metric	YOLOv8(KD)	YOLOv11(KD)	Difference
NANO version			
Precision	0.854	0.841	−0.013
Recall	**0.803**	0.786	−0.017
mAP@50	0.856	0.851	−0.005
Small version			
Precision	0.841	**0.912**	+0.071
Recall	0.799	0.785	−0.014
mAP@50	0.864	**0.883**	+0.019
Medium version			
Precision	0.894	0.875	−0.019
Recall	0.777	0.795	+0.018
mAP@50	0.864	0.864	+0.000
Large version			
Precision	0.906	0.878	−0.028
Recall	0.798	0.793	−0.005
mAP@50	0.881	0.866	−0.015

**Table 5:** COCO detection evaluation metric related to object size for fine-tuned YOLOv8(KD) and YOLOv8(KD) models of different sizes (s, m, l) tested on Düsseldorf test set. The best results are highlighted in bold.

Metric	YOLOv8(KD)-s	YOLOv8(KD)-m	YOLOv8(KD)-l	YOLOv11(KD)-s	YOLOv11(KD)-m	YOLOv11(KD)-l
mAP@0.5	0.864	0.864	0.881	**0.883**	0.864	0.866
AP_small	0.844	0.833	0.845	**0.858**	0.828	0.844
AP_medium	0.947	0.922	**0.964**	0.934	0.918	0.912
AP_large	x	x	x	x	x	x

## Data Availability

Data available in a publicly accessible repository (https://github.com/TheRomanFour/AbandonedLuggageDetection, https://app.roboflow.com/cars-0jbgu/luggage-person-detection-airport/, accessed on 29 April 2025).

## References

[1] Luna E., San Miguel J.C., Ortego D., Martínez J.M. (2018). Abandoned Object Detection in Video-Surveillance: Survey and Comparison. Intell. Sens..

[2] Kevin C. (2006). Smith and Pedro Quelhas and Daniel Gatica-Perez, ‘Detecting Abandoned Luggage Items in a Public Space’. https://infoscience.epfl.ch/handle/20.500.14299/47351.

[3] Smeureanu S., Ionescu R.T. Real-Time Deep Learning Method for Abandoned Luggage Detection in Video. Proceedings of the 6th European Signal Processing Conference (EUSIPCO).

[4] Santad T., Silapasupphakornwong P., Choensawat W., Sookhanaphibarn K. Application of YOLO Deep Learning Model for Real Time Abandoned Baggage Detection. Proceedings of the IEEE 7th Global Conference on Consumer Electronics.

[5] Chang J.Y., Liao H.H., Chen L.G. (2010). Localized Detection of Abandoned Luggage. EURASIP J. Adv. Signal Process..

[6] He K., Zhang X., Ren S., Sun J. (2017). Faster R-CNN: Towards real-time object detection with region proposal networks. IEEE Trans. Pattern Anal. Mach. Intell..

[7] Ultralytics (2025). Ultralytics Documentation. https://docs.ultralytics.com/.

[8] Qasim A.M., Abbas N., Ali A., Al-Ghamdi B.A.A.R. (2024). Abandoned Object Detection and Classification Using Deep Embedded Vision. IEEE Access.

[9] Redmon J., Divvala S., Girshick R., Farhadi A. (2016). You Only Look Once: Unified, Real-Time Object Detection. arXiv.

[10] Jocher G., Chaurasia A., Stoken A., Borovec J., Kwon Y., Michael K., Tao X., Fang J., NanoCode012, Imyhxy (2022). YOLOv5 and YOLOv8 Implementation Documentation. https://github.com/ultralytics/ultralytics.

[11] Panigrahi S., Nanda A., Swarnkar T. (2010). A Survey on Transfer Learning. IEEE Trans. Knowl. Data Eng..

[12] Liu S., Qi L., Qin H., Shi J., Jia J. (2021). A survey and performance evaluation of deep learning methods for small object detection. Expert Syst. Appl..

[13] Zhang Y., Sun P., Jiang Y., Yu D., Weng F., Yuan Z., Luo P., Liu W., Wang X. Bytetrack: Multi-object tracking by associating every detection box. Proceedings of the European Conference on Computer Vision.

[14] Padilla R., Netto S.L., Da Silva E.A. A Survey on Performance Metrics for Object-Detection Algorithms. Proceedings of the International Conference on Systems, Signals and Image Processing (IWSSIP).

[15] Xu H., Hu X., Zhou Y. Inter-Frame Multiscale Probabilistic Cross-Attention for Surveillance Object Detection. Proceedings of the IEEE 11th International Conference on Data Science and Advanced Analytics (DSAA).

[16] Carion N., Massa F., Synnaeve G., Usunier N., Kirillov A., Zagoruyko S. End-to-End Object Detection with Transformers. Proceedings of the European Conference on Computer Vision (ECCV).

[17] Sambolek S., Ivasic-Kos M. (2021). Automatic Person Detection in Search and Rescue Operations Using Deep CNN Detectors. IEEE Access.

[18] Sambolek S., Ivasic-Kos M. (2025). Person Detection and Geolocation Estimation in Drone Images. SN Compu. Sci..

[19] Chen H., Teng X., Su J., Li C., Hu C., Han M. (2025). Teacher Probability Reconstruction Based Knowledge Distillation within Intelligent Network Compression. Int. J. Intell. Netw..

